# Topics of Public Interest

**Published:** 1908-12

**Authors:** 


### TOPICS OF PUBLIC INTEREST.

### Medicolegal.

THE following is a decision of interest relating to the practice of Medicine, The People of the State of New York, respondent, v. John H. Woodbury Dermatological Institute, appellant.

1. Corporation Advertising to Practise Medicine—L. 1907, ch. 344, sec. 15. A corporation organised under the provisions of L. 1848, ch. 40, authorising the formation of corporations for manufacturing, mining, mechanical or chemical purposes, which advertises to practise medicine, may be convicted under the provisions of L. 1907, ch. 344, sec. 15, providing that "any person not registered as a physician who shall advertise to practise medicine, shall be guilty of a misdemeanor."

2. Hospitals, infirmaries and similar institutions—Words "any person" include corporation. While the words "any person" include a corporation as well as an individual under section 5 of the Statutory Construction Law (L. 1892, ch. 677), they were nevertheless not intended to apply to hospitals, infirmaries and similar institutions organised under the provisions of section 80 of the Membership Corporations Law (L. 1895, ch. 559, as amended by L. 1900, ch. 404) which have legislative authority for the practice of medicine.

People v. Woodbury Dermatological Inst., 124 App. Div., 877, affirmed. (Argued June 1, 1908; decided September 29, 1908.)

Appeal from an order of the Appellate Division of the Supreme Court in the first judicial department, entered March

20, 1908, which affirmed a judgment of the court of special sessions of the city of New York convicting the defendant of the misdemeanor of unlawfully advertising to practise medicine without authorisation and registration. The facts, so far as material, are stated in the opinion.

Roswell S. Nichols for appellant. The defendant did not advertise to practise medicine within the meaning of the act. (S. E. M. Inst. v. State, 103 N. W. Rep., 1078; S. E. M. Inst. v. Platner, 103 N. W. Rep., 1079; Underwood v. Scott, 32 Pac. Rep., 942; People v. Allcutt, 117 App. Div., 546.) It was not the intention of the legislature to include corporations when it used the word "person" in chapter 344 of the Laws of 1907. (Manhattan Co. v. Kaldenberg, 165 N. Y., 1; Burke v. Basso, 180 N. Y., 341; Smith v. B. & A. R. R. Co., 99 App. Div., 94; 181 N. Y., 132; H. B. Co. v. Hand, 104 App. Div., 390; Pharmaceutical Society v. L. & P. S. Co., L. R. (5 App. Cas.), 857; O'Duffy v. Jaffe, Surgeon Dentist, Ltd., L. R. (2 Ir. Rep., 1904), 27; Parks, Gunston & Lee v. Ward, L. R. (2 K. B. Div.), 1; Beastone v. F. Bank. 12 Pet., 102). Section 5 of the Statutory Construction Law does not require the courts to construe the word "person" to include a corporation, where it appears that the legislature did not so intend. (People v. U. Ins. Co., 15 Johns., 358; Stack v. City of Brooklyn, 150 N. Y., 335; Spencer v. Meyer, 150 N. Y., 269; People ex rel Wood v. Lacombe, 99 N. Y., 43; Dallman v. Moore, 70 Miss., 267.)

William Travers Jerome, District Attorney (Robert S. Johnstone of counsel), for respondent. The word "person" in the statute includes a corporation. (L. 1907, ch. 344; Dent v. West Virginia, 129 U. S., 114; Ottaway v. Lowden, 172 N. Y., 129; People ex rel. Armstrong v. Warden, 183 N. Y., 223; O'Brien v. Mayor, etc., 139 N. Y., 548; Risley v. Phœnix Bank, 83 N. Y., 318; P. Society v. Supply Co., 5 App. Cas., 857; Brown v. Mayor, etc., 66 N. Y., 385; Hannon v. S. C. Co., 167 N. Y., 244; Deaton v. Lawson, 82 Pac. Rep., 879; Matter of Neagle, 139 U. S., 1; People v. Girard, 145 N. Y., 105.

Willard Bartlett, J. The statute now in force which regulates the practice of medicine in this state provides "any person not a registered physician who shall advertise to practice medicine, shall be guilty of a misdemeanor". (Laws of 1907, chap. 344, Sec. 15.)

The district attorney of the county of New York laid an information before the Court of Special Sessions, accusing the defendant of the crime of unlawfully advertising to practise medicine in violation of this prohibition. The defendant was convicted upon proof sufficient to establish the fact that it had so advertised. It was contended before the trial court, however, and it is contended here, that a corporation is not chargeable with liability under the statute because the word "person" as used therein, cannot be held to apply to a corporation at all, but is exclusively applicable to an individual human being. The prosecution answered this proposition by reference to the Statutory Construction Law (Laws of 1892, chap. 677, sec. 5) which provides that "the term person includes a corporation and a joint stock association." The defendant replied that the provision quoted from section 5 of the Statutory Construction Law is qualified by the limitation contained in Section 1 of the same act, which declares that the chapter is "applicable to every statute unless its general object or the context of the language construed, or other provisions of law indicate that a different meaning or application was intended from that required to be given by this chapter." The argument of the defendant is that the context in the general act of 1907, regulating the practice of medicine, which contains the prohibition against advertising to practise medicine by any person, not a registered physician, indicates conclusively that "any person" therein mentioned could not possibly have been intended to mean "any corporation." The Court of Special Sessions, however, and the Appellate Division (with no dissenting judge) reached a contrary conclusion, holding that artificial persons as well as natural persons fell within the prohibition of the statute. Mr. Justice Ingraham, who wrote one of the opinions for the majority of the Appellate Division, called attention to the fact, that the provision in the Statutory Construction Law to the effect that the term person includes a corporation, was taken from 718 of the Penal Code, and section 955 of the Code of Criminal Procedure, which were repealed by section 37 of the Statutory Construction Law itself. This substitution and repeal, seemed to him clearly indicative of the intention of the legislature to make the provision of the Statutory Construction Law, applicable to all cases in which a corporation had committed an act which by law was made a criminal offense if committed by a natural person.

The only difficulty involved in the adoption of this view grows out of the existence of hospitals, dispensaries and similar corporate institutions which are unquestionably authorised by law to practise medicine—although, of course, only through the agency of natural persons who are duly registered as physicians. The defendant was incorporated under the act of February 17, 1848 (L. 1848, ch. 40), to authorise the formation of corporations for manufacturing, mining, mechanical or chemical purposes, and the objects for which it was formed were stated in the certificate of incorporation as follows: "The carrying on of business of manufacturing chemical preparations and printing, publishing and selling books and pamphlets relating to the same and advertising same." There could be no suggestion or pretense that this was a hospital corporation or dispensary. In reference, however, to hospitals and dispensaries it could hardly have been the intention of the legislature in the act of 1907, to prohibit such corporations from advertising to do what they might do lawfully—that is to say, from advertising to practise medicine; yet it is argued that if we hold that the act of 1907, makes it a misdemeanor for any corporation (making the term "person" embrace a corporation) to advertise to practice medicine, we must also hold that incorporated hospitals and dispensaries fall within the prohibition. If this were the necessary result of the construction adopted in the courts below, I think it would furnish a strong reason for rejecting that construction.

It seems to me, however, that we can affirm this judgment without in any wise denying the lawful right of hospitals, dispensaries and similar corporate institutions to advertise their readiness to exercise their lawful functions; and this simply for the reason that the general medical law of 1907, is obviously not intended to apply to the case of such corporations at all. In other words, the prohibitions therein contained against the practice of medicine, without lawful registration in this state or in violation of any of the provisions of the statute or against advertising by any person not a registered physician, were not intended to apply and plainly could not reasonably be held to apply to corporate bodies which by the express provisions of other statutes, are authorised to carry on the practice of medicine upon compliance with their provisions and without registration.

The incorporation of hospitals is provided for in section 80 of the Membership Corporations Law (L. 1895, ch. 559, as amended by L. 1900, ch. 404). Five or more persons may become a corporation for the purpose of erecting, establishing or maintaining "a hospital, infirmary, dispensary, or home for invalids, aged, or indigent persons," by making the prescribed certificate and obtaining the written approval of the state board of charities and a justice of the Supreme Court of the district in which the principal office or place of business of the corporation is to be located. The statute expressly provides that "the systems of medical practice or treatment to be used or applied in such hospitals, infirmary, dispensary or home" may be specified in the certificate. Thus, a hospital duly incorporated under the Membership Corporations Law unquestionably holds itself out as being able to diagnosticate, treat, operate and prescribe for human disease, pain, injury, deformity or physical condition; and such corporations do in fact offer and undertake publicly and frequently through the agency of advertisements to diagnosticate, treat, operate and prescribe for such diseases. An institution of this character, possessing legislative authority to practise medicine by means of its staff of registered physicians and surgeons, comes under the direct sanction of the law in so doing, and by the plainest implication under well-settled rules of statutory construction relating to enactments dealing with the same general subject-matter, are excepted from the operation of the act of 1907, under which the defendant was convicted.

It is suggested in behalf of the appellant that to attribute to the legislature a design to prohibit a corporation which is not registered as a physician from advertising to practise medicine, is to charge the lawmakers with doing an absurd act, inasmuch as it is impossible under the law for a corporation to register as a physician. There is nothing in this point. It might just as well be urged that there is no need of a law prohibiting a minor from voting, since everybody knows that a minor has no legal right to vote. This fact does not prevent illegal attempts on the part of minors to exercise the right of suffrage, and the facts in the present case show that a corporation may undertake to practise medicine without authority of law.

Believing as I do, that the construction of section 15 of Chapter 344 of the Laws of 1907, adopted by the courts below, may be sustained without affecting the rights of incorporated hospitals or dispensaries, I think the judgment of conviction was correct and should be affirmed.

Culle, Ch. J., Gray, Haight, Werner, Hiscock and Chase, JJ., concur. Judgment of conviction affirmed.

People v. Woodbury Dermatological Inst., 192 N. Y., Rep., 454; Adv. Sheets No. 409, October 31, 1908.

---

W. E. Deeks of Ancon, Panama, gives four types of malarial fever as seen on the Isthmus of Panama, although the quartan is not endemic there. These types differ microscopically in the development of crescents or not, in their life history, in perniciousness, and in their course. Most of the malaria is curable by quinine. Rarely it affects the brain with few constitutional symptoms present. In cerebral cases quinine is given hypodermically in twenty grain doses, every two hours, up to sixty or eighty grains of the hydrochlorate of quinine and urea. Post-febrile toxemia is common and important. Forty per cent. of cases develop albuminuria which in general clears up, but when this is not the case results in a chronic interstitial nephritis. Kidney symptoms and very low tension pulse indicate acute toxemia. This is more often the case in the colored than in the white race. —*Medical Record*, November 21, 1908.

